# Prevalence of adjustment disorder among cancer patients, and the reach, effectiveness, cost-utility and budget impact of tailored psychological treatment: study protocol of a randomized controlled trial

**DOI:** 10.1186/s40359-019-0368-y

**Published:** 2019-12-23

**Authors:** Florie E. van Beek, Lonneke M. A. Wijnhoven, Femke Jansen, José A. E. Custers, Eline J. Aukema, Veerle M. H. Coupé, Pim Cuijpers, Marije L. van der Lee, Birgit I. Lissenberg-Witte, Ben Wijnen, Judith B. Prins, Irma M. Verdonck-de Leeuw

**Affiliations:** 1 Department of Clinical, Neuro & Developmental Psychology, Amsterdam Public Health Research Institute, Vrije Universiteit Amsterdam, Amsterdam, The Netherlands; 2 Department of Medical Psychology, Radboudumc Nijmegen, Radboud Institute of Health Sciences, Nijmegen, The Netherlands; 3 Ingeborg Douwes Centrum, Centre for Psycho-Oncology, Amsterdam, The Netherlands; 4 Department of Epidemiology and Biostatistics, Amsterdam UMC, Vrije Universiteit Amsterdam, Amsterdam, The Netherlands; 5 Helen Dowling Institute for Psycho-Oncology, Utrecht, The Netherlands; 6 Trimbos Institute, Utrecht, The Netherlands; 7 Department of Otolaryngology-Head and Neck Surgery, Amsterdam UMC, VUmc, Cancer Center Amsterdam, Amsterdam, The Netherlands

**Keywords:** Psychological intervention, Cancer patients, Psychological distress, Adjustment disorder, Randomized controlled trial

## Abstract

**Background:**

Information on the prevalence of adjustment disorders among cancer patients and the value of psychological interventions in this group of patients is limited. This study investigates the prevalence of adjustment disorders among cancer patients as well as the reach, effectiveness, cost-utility and budget impact of a tailored psychological intervention.

**Method:**

This study consists of two parts. Part 1 is an observational study among a representative group of mixed cancer patients after cancer treatment on the prevalence of adjustment disorder as well as the uptake (i.e. reach) of psychological treatment. In Part 2, patients diagnosed with an adjustment disorder are invited to participate in a randomized controlled trial. Patients will be randomized to the intervention (access to the tailored psychological intervention) or control group (waitlist period of 6 months). The psychological intervention consists of three modules: one module containing psycho-education (3 sessions, all patients) and two additional modules (maximum of 6 sessions per module) provided as continuum, in case needed. Module 2 and 3 can consist of several evidence-based interventions (e.g. group interventions, mindfulness, eHealth) The primary outcome is psychological distress (HADS). Secondary outcomes are mental adjustment to cancer (MAC) and health-related quality of life (EORTC QLQ-C30). To assess the cost-utility and budget impact, quality of life (EQ-5D-5 L) and costs (iMCQ and iPCQ) will be measured. Measures will be completed at baseline and 3 and 6-months after randomization.

**Discussion:**

This study will provide data of the prevalence of adjustment disorders and the reach, effectiveness, cost-utility and budget impact of a tailored psychological intervention.

**Trial registration:**

Netherlands Trial Register identifier: NL7763. Registered on 3 June 2019.

## Background

Worldwide the incidence of cancer is growing. It was estimated that 18.1 million people worldwide were newly diagnosed with cancer in 2018 [1]. There is convincing empirical evidence that cancer patients have to deal with a wide range of physical symptoms and psychological, social and existential problems related to cancer and its treatment, both during treatment and at (long-term) follow-up. Psychological problems involve symptoms related to anxiety and depression, but also problems with adjustment to cancer and its sequelae [2]. In case of severe and persistent problems with adjustment to cancer, an adjustment disorder can be diagnosed according to the Diagnostic and Statistical Manual of Mental Disorders (DSM-V) [3]. An adjustment disorder is characterized by symptoms such as anxiety, depression or fatigue and can be developed in case of insufficient protective factors (e.g. resilience or meaning making), which can result in significant impairments in a patients’ life (e.g. work or study, social relations or emotional problems) [3].

Earlier studies showed prevalence rates of adjustment disorder varying from 6% to over 19%, as measured using diagnostic interviews. In a meta-analysis of Mitchell et al. (2011) [4] the prevalence of adjustment disorder among cancer patients was estimated to be 19%, while in more recent studies somewhat lower prevalence rates of 6 to 17% were reported [5–8]. In a recent large study in Germany of Mehnert et al. (2014) [7] in a population of mixed cancer patients who had their cancer diagnosis for on average 13.5 months, 11% of all patients had an adjustment disorder in the previous 4 weeks (independently of other psychological disorders such as anxiety or depression).

In case an adjustment disorder is diagnosed, evidence or practice based psychological interventions should be available and provided to the patient [3]. However, in clinical practice, psychological treatment is often not optimally accessible for cancer patients, especially for those with an adjustment disorder. Several bottlenecks in the organization of psychological treatment have been identified, including problems with identifying cancer patients with an adjustment disorder, and problems with referral to psychological treatment [9–13]. In addition, when an adjustment disorder is diagnosed in cancer patients, the accessibility of psychological care is limited, since there is currently no adequate coverage and reimbursement of adjustment disorders treatments in cancer patients after active cancer treatment [14].

A systematic review of Faller et al. (2013) [15] and several studies published after the conduction of this review [16–24] showed evidence for the effectiveness of psychological interventions targeting cancer patients, including self-management interventions, eHealth interventions, group interventions, and individual interventions. Also, two reviews showed that psychological interventions targeting cancer patients are likely to be cost-effective at potentially acceptable willingness-to-pay thresholds [25, 26]. Three recent cost-utility studies, on meaning-centred group psychotherapy, stepped psychological care, and blended cognitive behavioural therapy, even showed that psychological treatment is more effective and potentially less costly compared to care-as-usual [16, 27, 28]. However, to the best of our knowledge, no study specifically focused on the effectiveness, cost-utility and budget impact of psychological interventions in cancer patients with an adjustment disorder.

This randomized controlled trial, therefore, aims to provide insight into the prevalence of an adjustment disorder among cancer patients, and the reach, effectiveness, cost-utility and budget impact of a tailored psychological intervention. The results are relevant to improve care (including accessibility and reimbursement) for cancer patients with an adjustment disorder.

## Methods

The methods section of this study protocol is written in accordance with the STROBE statement for cohort studies and CONSORT statement for reporting randomized controlled trials (RCT) [29, 30].

### Study design

**Fig. 1:**
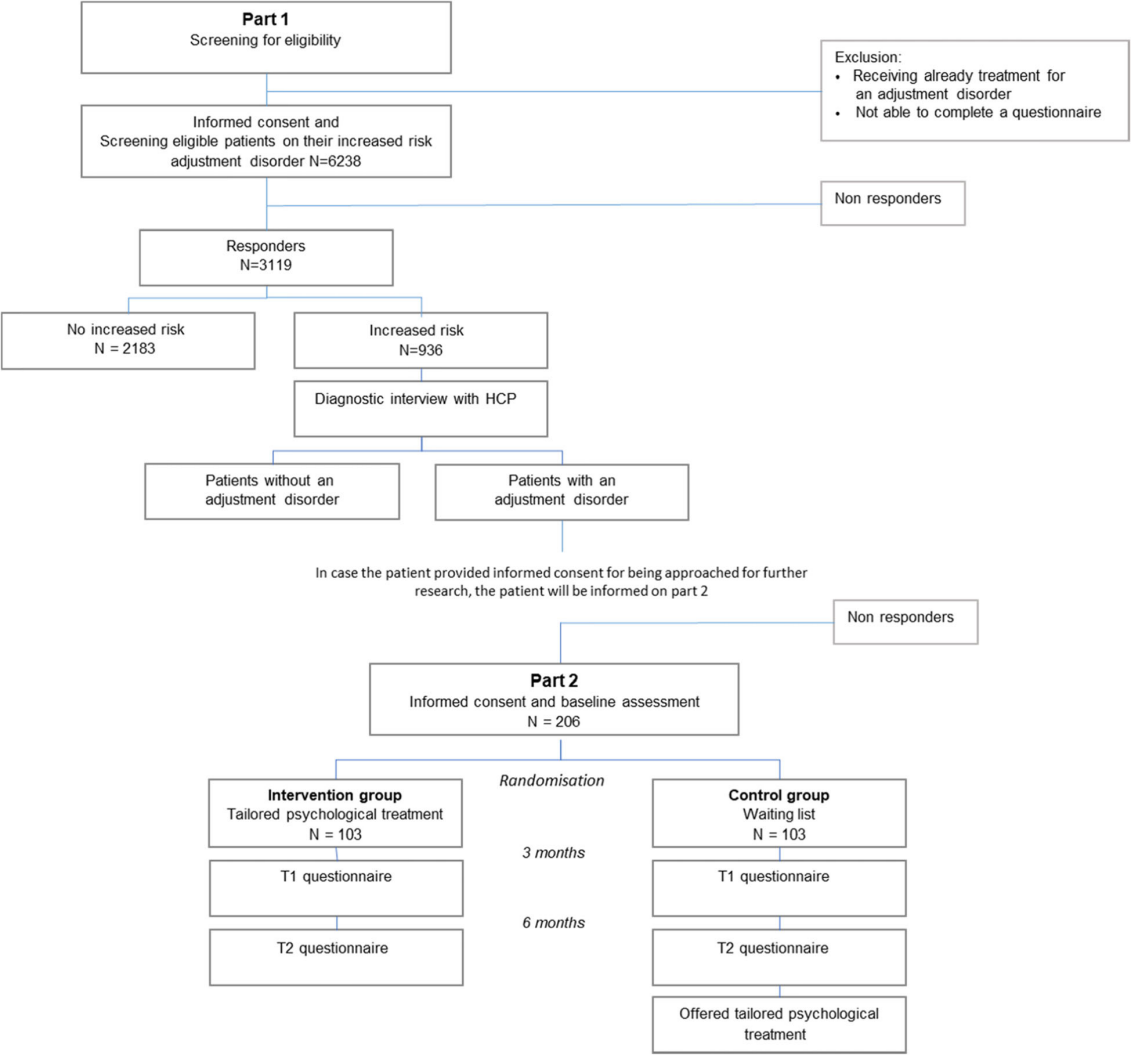
Study design of part 1 and 2 combined with expected number of patients per step

**Fig. 2:**
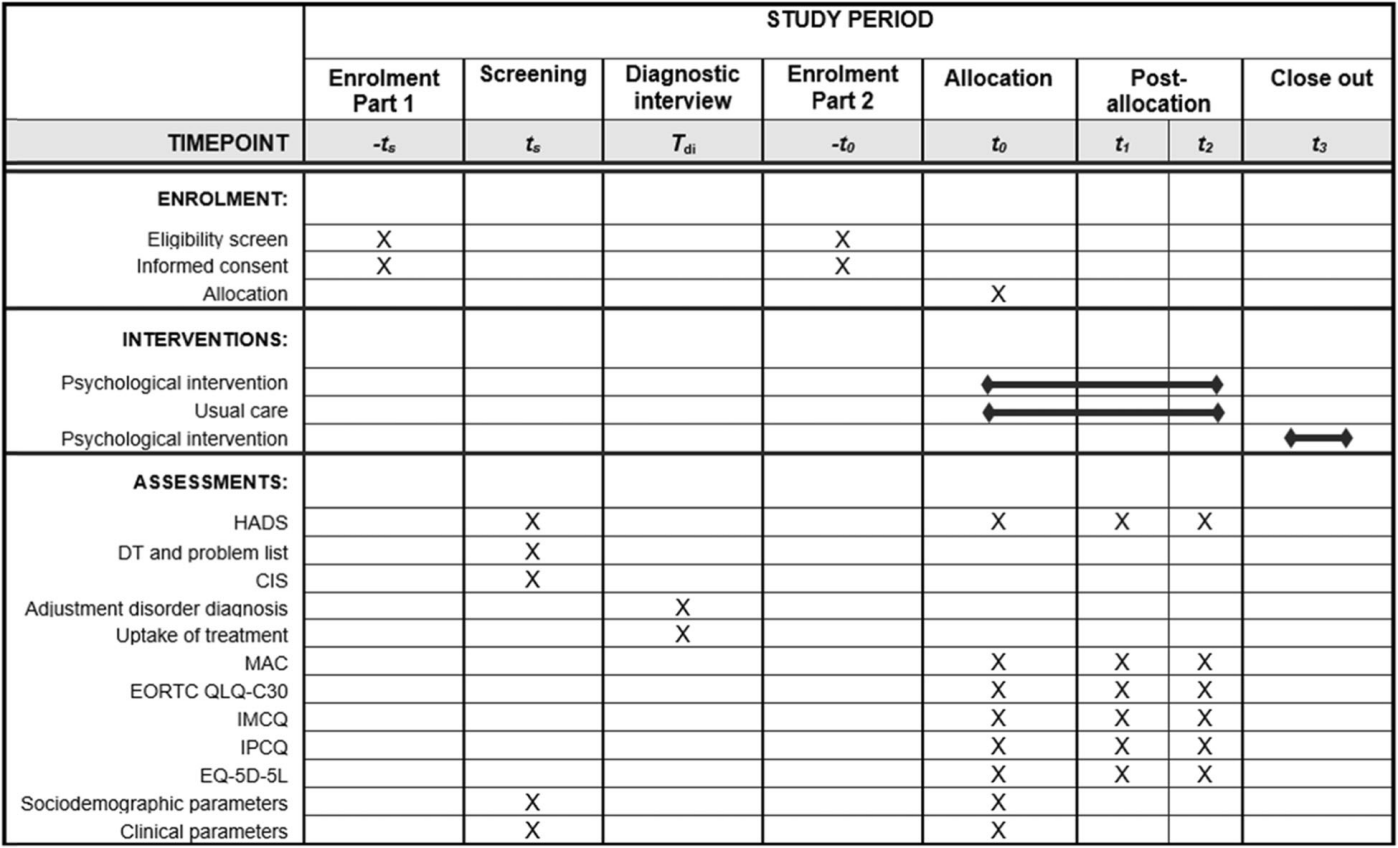
Standard protocol items as highly recommended according the SPIRIT

This study consists of two parts. Part 1 is an observational study among a representative group of cancer patients after medical treatment in which the prevalence of an adjustment disorder as well as the uptake of psychological treatment (i.e. reach) is assessed. Part 2 includes an RCT in which the effectiveness, cost-utility and budget impact of a tailored psychological intervention compared to waitlist control care is investigated. The patient flow through the study is shown in Fig. 1 and the schedule of enrolment, assessments and interventions is provided in Fig. 2. This study has been approved by the Medical Ethical Committee of the VU University Medical Center.

### Part 1: study population and inclusion procedure

For Part 1 of this study, we aim to screen a representative group of mixed cancer patients on the prevalence of an adjustment disorder. Participants will be included in this study in case they are diagnosed with cancer (all types and stages, except non-melanoma skin cancer) before July 2018, finished cancer treatment with curative or palliative intent (all treatment modalities, except for endocrine therapy in breast/prostate cancer) and are aged ≥18 years. A random selection will be drawn by the Netherlands Cancer Registry (NCR) of patients from participating departments of participating hospitals. The NCR registers all newly diagnosed cancer patients within 6 months after diagnosis in the Netherlands. The patient information letter will be sent by post to the eligible patient by the former treating physician. When a patient is willing to participate, he or she is asked to provide informed consent. All data will be collected using the Patient Reported Outcomes Following Initial treatment and Long-term Evaluation of Survivorship (PROFILES) system. PROFILES is a registry and is directly linked to data of the NCR. All necessary permissions were obtained to access and use the data and who gave this permission.

### Part 1: prevalence and reach

The prevalence of adjustment disorder diagnosis will be investigated using a two-step approach. First, patients will be screened on the increased risk for an adjustment disorder using a set of screening questionnaires. Patients will be asked to complete these questionnaires online or via paper-and-pencil. The screening questionnaires consist of the distress thermometer (DT), the problem list and the Hospital Anxiety and Depression Scale (HADS). The DT measures the level of distress experienced in the last week on a thermometer ranging from 0 (no distress) to 10 (extreme distress) [31]. The problem list measures 47 different problems, including practical problems, family/social problems, emotional problems, spiritual/religious concerns, physical problems and a free-text section on any additional problems on a dichotomous scale (Yes/No), as well as a single item on wanting to talk to a psychologist, psychotherapist or psychiatrist. All items refer to ‘last week’. The HADS includes 14-items measuring psychological distress (HADS-T), anxiety and depression as further discussed below [32]. In addition, patients will be asked to complete the Checklist Individual Strength (CIS) and questions on sociodemographic (e.g. marital status, living situation, education level, employment status) and clinical characteristics (e.g. tumour recurrence). The CIS consists of 20 items (7-point Likert scale) on subjective experiences of fatigue, concentration, motivation, and physical activity [33]. The CIS which strongly resembles the multidimensional fatigue inventory (MFI) [34], has been shown to be a valid and reliable measurement to investigate fatigue with good internal consistency [35].

Second, patients with an increased risk for an adjustment disorder (i.e. HADS score > =11, DT > =4 or work/school/study problems, family or social problems, emotional problems, fatigue or wanting to talk to a psychologist or social worker as reported on the problem list) will be invited for a diagnostic interview by a registered psychologist, psychotherapist or psychiatrist trained in clinical care for cancer patients with an adjustment disorder (further called healthcare professional (HCP)). During this diagnostic interview by telephone or face to face the presence of an adjustment disorder will be investigated. HCPs will follow the Dutch guideline on adjustment disorder diagnosis [36]. The guideline committee recently defined adjustment disorder in patients with cancer as the combination and interaction among three pillars, namely stressors (e.g. cancer diagnosis, fear of cancer recurrence, physical changes in a patients’ appearance), insufficient protective factors (e.g. resilience, physical health, meaning, social support, autonomy), and the experience of symptoms (e.g. anxiety, depression, fatigue, relation problems, limited work productivity) [3]. During the diagnostic interview, the HCP will also complete a form on sociodemographic (age, gender) and clinical (tumour site, stage, phase of cancer (acute/chronic/palliative), time since diagnosis, treatment modality) parameters, and a form on stressors experienced, protective factors, symptoms experienced and actual psychologic diagnosis. To monitor the robustness of this diagnosis, the diagnostic interview will be audio recorded in case the patient provides specific informed consent on this matter. Approximately 5 % of the total diagnostic interviews will be scored twice (adjustment disorder yes/no).

Additionally, all participating HCPs will be asked to complete a questionnaire on type of care (primary or secondary care), profession and training, and years of experience in working with cancer patients.

### Part 2: study population and inclusion procedure

Patients diagnosed with an adjustment disorder in Part 1 of this study will be invited to participate in Part 2 (the RCT). Patients will be first introduced to the study by the HCP. The coordinating researcher of the study will further inform interested patients by phone. Also, the patient information form and informed consent of Part 2 will be sent to the patient. After obtaining informed consent, the patient is asked to fill in the baseline questionnaire via the internet or using paper-and-pencil. After completing the baseline questionnaire, the patient will be randomized into either the intervention group (start of the tailored psychological intervention within 3 a 4 weeks) or the waitlist control group (receive a tailored psychological intervention after a waitlist period of 6 months). All patients will be asked to complete questionnaires before randomization (T0), and 3 (T1) and 6 months (T2) after randomization. In case of non-response to these questionnaires a reminder letter and a paper-and-pencil questionnaire will be sent after 3 weeks. If they do not respond to this reminder, they will be contacted by telephone within 2 weeks. Reasons for dropouts will be registered. Data will be collected using the PROFILES system.

### Part 2: tailored psychological intervention

The psychological intervention investigated in this proposed project follows the Dutch guideline on diagnosis of adjustment disorders [36] and consists of 3 modules. Module 1 encompasses a maximum of 3 sessions on psycho-education with an HCP. Module 2 and 3 encompass both a maximum of 6 sessions. Module 2 and 3 can consist of all evidence-based interventions outlined in the guideline on adjustment disorders, such as cognitive behavioural therapy, mindfulness, group interventions, online interventions or pharmacotherapy [3]. Depending on the wishes and needs of the patient a specific type of therapy per module will be offered to the patient (tailored treatment) [37].

After each last session of a module the HCP will assess in accordance with the patient if another module is needed. To support this assessment the patient will complete the HADS during this session, following the guideline on diagnosis of adjustment disorders [36]. When sufficiently effective, only the short psychological treatment module 1 will be provided. The longer treatments (module 2 or module 3) will only be offered if the previous psychological treatment module was insufficiently effective, so the 3 modules will be provided as a continuum.

### Part 2: control group

Patients randomised to the waitlist control group receive the tailored psychological intervention after a waitlist period of 6 months. This period is comparable with the usual waitlist period for psychological care in the clinical practice. During the waitlist period it is allowed to receive usual care. Usual care received during the study will be measured using the healthcare utilization questionnaire discussed below.

### Part 2: randomization

Randomization will be conducted centrally by an independent person in blocks of four and six using an automatically created randomisation list. Randomization will be stratified for the patient self-reported prognosis and severity of psychological distress by an independent person. Patients are not blinded to treatment allocation. Data managers will be blinded to the treatment allocation.

### Part 2: outcome assessments

The primary outcome of Part 2 is psychological distress. Secondary outcomes are Mental Adjustment to Cancer and health-related quality of life. In addition, the cost-utility and budget impact of the psychological intervention will be investigated (Table 1). To determine cost-utility and budget impact, Quality-Adjusted Life-Years (QALYs) and costs will be measured. For the cost-utility and budget impact analyses from a societal perspective, intervention costs, healthcare costs, costs of the patient and his/her family (e.g. informal care costs and travel costs) and costs in other sectors (e.g. productivity losses) will be collected. The healthcare perspective will only include intervention costs and healthcare costs and the insurer perspective will only include costs reimbursed by the healthcare insurance company.

**Table 1:** Outcome measures and used instruments

Part	Outcome	Outcome measure	Instrument
1	Primary outcome	Reach: prevalence of adjustment disorder (yes/no) and uptake of the tailored psychological intervention (yes/no)	Screening (DT, problem list and HADS) and interview
1	Other collected measurements	Fatigue	CIS
2	Primary outcome	Psychological distress	HADS
2	Secondary outcomes	Mental adjustment to cancer	MAC
		Health-related quality of life	EORTC-QLQ-C30
		Cost-utility measures	
		Medical utilization costs Informal care costs	iMCQ questionnaire iMCQ questionnaire
		Productivity losses	iPCQ questionnaire
		Quality adjusted life-years	EQ-5D-5 L
1–2	Other collected measurements	Socio-demographic and clinical characteristics	Study specific questionnaire
		Healthcare professional characteristics	Short questionnaire on type of care

*HADS* Hospital Anxiety and Depression Scale, *CIS* Checklist Individual Strength, *MAC* Mental Adjustment to Cancer, *EORTC QLQ-C30* European Organization for Research and Treatment of Cancer Quality of Life Questionnaire, *iMCQ* Medical consumption questionnaire, *iPCQ* Productivity cost questionnaire, *HCP* healthcare professional

#### Primary outcome measures

Psychological distress will be measured with the HADS. The HADS is a 14-item (4-point Likert scale) patient-reported outcome measure for measuring psychological distress, anxiety and depression [32]. All items refer to the last week. The total HADS (HADS-T) score ranges from 0 to 42. A higher score indicates higher levels of distress. The HADS is a valid instrument for use in cancer patients and Dutch persons [38].

#### Secondary outcome measures

Cognitive and behavioural response to cancer diagnosis and treatment will be assessed using the 40-item (4-point Likert scale) Mental Adjustment to Cancer (MAC) questionnaire. The MAC scale comprises five subscales: fighting, spirit, helplessness/hopelessness, anxious preoccupation, fatalism and avoidance [39]. All items refer to the current situation. A higher score on the subscales indicate more fighting spirit, helplessness/hopelessness, anxious preoccupation, fatalism or more avoidance [40, 41]. Besides, based on these five sum scores, two summary scores can be calculated, namely positive adjustment (17 items) and negative adjustment (16 items). Psychometric characteristics of the MAC have previously been investigated among mixed cancer patients, including Dutch cancer patients [41].

Health-related quality of life will be measured with the 30-item (4-point Likert scale) European Organization for Research and Treatment of Cancer Quality of Life Questionnaire Core (EORTC QLQ-C30). This questionnaire consists of a global health-related quality of life scale, five functional scales (physical functioning, role functioning, emotional functioning, cognitive functioning and social functioning), three symptom scales (nausea and vomiting, fatigue and pain) and 6 single items relating to dyspnoea, insomnia, loss of appetite, constipation, diarrhoea and financial difficulties [42, 43]. All scales and single items can be converted to a score from 0 to 100. A higher score on the functioning scales or the global quality of life scale represents a better quality of life, whereas a higher score on the symptom scales or the single items indicate a higher level of symptoms. The EORTC QLQ-C30 is a valid and reliable instrument for health-related quality of life assessments in various cancer populations [42, 43].

Other outcomes as socio-demographic and clinical parameters and HCP characteristics are similar to the already collected measurements in Part 1 of this study.

#### Outcome measures on cost-utility and budget impact

Costs will be measured by questionnaires developed by the Institute for Medical Technology Assessments of the Erasmus University Rotterdam, as recommended in the Dutch Health Care Insurance Board (CVZ) guideline [44]. Healthcare utilization (e.g. visits to the general practitioner, visits to the medical specialist, and hospitalization) and received informal care will be assessed with the Medical Consumption Questionnaire (iMCQ) [45]. Losses due to absenteeism and presenteeism (decreased work productivity) will be assessed with the productivity cost questionnaire (iPCQ) [46]. In this study an adapted version of both questionnaires will be used with a recall period of 3 months.

QALYs will be calculated by multiplying the time spent in a specific health state with the quality (utility) of that health state. Utilities will be measured by using the EuroQol 5-demensions 5-item (5-point Likert scale) instrument (EQ-5D-5 L). The EQ-5D-5 L consist of five dimensions of quality of life (mobility, self-care, usual activities, pain/discomfort and anxiety/depression) [47]. The resulting profile of answers can be transformed to a value given by the general public: the EQ-5D index using the Dutch index tariff [48]. A visual analogue scale is also included, which represents the patients’ judgment of his own health state on a scale from 0 (worst health state) to 100 (best health state).

### Sample size

The sample sizes of Part 1 and 2 depend on each other. To demonstrate an effect size in Part 2 of 5 points on the HADS as statistically significant, anticipating a standard deviation of 11 (i.e. two times the baseline standard deviation of the HADS), 77 participants in each condition are needed at follow-up (power 80%, significance level 5%). Anticipating a drop-out rate of 25% between baseline and 6 month follow-up, 103 participants per condition, thus 206 in total, need to be included at baseline. Estimating a willingness to participate in this RCT of 60%, 343 patients will need to be approached to participate in the RCT in Part 2 [49]. Taking into account a prevalence rate of adjustment disorders of 11% as estimated by Mehnert et al. [7], 3119 cancer patients need to be screened in Part 1 to identify 343 patients with adjustment disorders. Anticipating a response rate of 50%, 6238 patients need to be approached for the screening.

### Statistical analysis

In Part 1 and Part 2 quantitative analyses will be performed using the IBM Statistical package for the Social Science (SPSS) version 25 (IBM Corp., Armonk, NY USA) and STATA version 14 Descriptive statistics will be generated for all socio-demographic and clinical characteristics and outcome measures. Chi-square tests, independent t-tests and Mann-Whitney tests (in case of non-normality of the measure) will be used to analyse whether randomization resulted in comparable patient groups. Analyses will be performed according to the intention-to-treat principle. A *p*-value < 0.05 will be considered significant.

Part 1 aims to investigate the prevalence of having an adjustment disorder, to investigate the uptake of psychological treatment among cancer patients with an adjustment disorder and to investigate its determinants. Determinants of having an adjustment disorder or uptake of psychological care will be entered one-by-one to the logistic regression model using a p-value for entry of 0.05. Potential determinants include scores on the patient-reported outcomes, socio-demographic, and clinical characteristics of the patient, as well as HCP characteristics. Part 2 aims to investigate the effectiveness of the intervention on the primary outcome measure (HADS) with the use of linear mixed models. The linear mixed model will contain a fixed effect for arm/group, time and their two-way interaction, and a random effect for subject. A significant (*p* < 0.05) two-way interaction indicates a difference in effectiveness between the intervention and the control group. In that case, post-hoc effect sizes, at 3 and 6 months follow-up, will be calculated using Cohen’s d.

For the cost-utility analyses, pertinent guidelines will be used [44]. Analyses will be conducted in agreement with the intention-to-treat principle from both a societal and healthcare perspective. Costs will be calculated by multiplying resource use by integral cost prices as presented in the cost guideline [44]. Productivity losses due to absenteeism and presenteeism will be calculated using the friction cost approach. Missing data on costs and utilities will be imputed using multiple imputation. The time horizon will be set at 6 months, and therefore neither costs nor effects need to be discounted. Incremental cost-utility ratios (ICURs) will be calculated with their 95% confidence intervals using 5000 bootstrap replications, which will be projected on a cost-utility plane. In addition, ICUR acceptability curves will be presented and sensitivity analyses will be performed focusing on uncertainty surrounding most important cost items.

For the budget impact analyses, the current guideline on budget impact analyses of the Dutch National health Care Institute (ZIN) and the International Society for Pharmacoeconomic and Outcomes Research will be used [44, 50]. To perform a budget impact analysis insight is needed on: a) size of the target population, b) costs of the intervention and 3) other costs (such as other healthcare costs). Several budget impact analyses will be performed, as this will provide insight into the uncertainty surrounding the budget impact of providing psychological treatment to patients with cancer with an adjustment disorder. Budget impact analyses will be performed from a healthcare (i.e. including intervention costs and healthcare costs), societal (i.e. including intervention costs, healthcare costs, costs of the patient and his/her family and costs in other sectors) and insurer perspective (i.e. including costs reimbursed by the healthcare insurance company).

## Discussion

This paper describes the protocol of a study that aims to provide evidence on the prevalence of an adjustment disorder among cancer patients and the reach, effectiveness, cost-utility and budget impact of tailored psychological treatment.

The first part of this project aims to investigate the prevalence. As mentioned above, previous studies have found prevalence rates varying between 6 and 19% [5–8]. However, these studies were heterogeneous in terms of cancer type and methodological quality, warranting further research. Also, the conceptualization of adjustment disorder among cancer patients is poorly studied, which may limit the precision of adjustment disorder diagnosis [51]. Most studies used diagnostic interviews to diagnose adjustment disorder [5–8]. Mehnert et al. [7], on the other hand, first screened patients on their level of psychological distress using the patient health questionnaire (score of 9 or higher on the PHQ-9), followed by a diagnostic interview in those patients with increased levels of psychological distress. As it is estimated not to be feasible to conduct a diagnostic interview in the more than 3000 patients needed for this study, we will in line with the study of Mehnert et al. [7] first screen the patients on their risk for having an adjustment diagnosis using patient-reported outcomes measures. As it is not yet clear which screening questionnaire should be used to preselect patients on their risk for having an adjustment disorder [3], we will preselect patients using the DT, problem list and the HADS. Previous studies have investigated the predictive value of the DT [52–54], HADS [53, 55–59], PHQ-2 [52], Zung Self-rating Depression Scale (ZSDS) [60] and One Question Interview (OQI) [54] to identify patients with an adjustment disorder. Most studies, however, have been conducted on the HADS and DT. Three studies that investigated the predictive value of the DT showed that a cut-off ranging between the > 3 and > 5 resulted in the best screening performance, whereas other studies investigating the HADS found a cut-off ranging between > 9 and > 15. In our study all patients with an increased risk on the questionnaire (i.e. HADS score > =11, DT > =4, certain problems on the problem list or wanting to talk to a psychologist or social worker) will be asked to participate in the diagnostic interview. In line with the study of Mehnert et al. [7], we expect to find a prevalence rate of adjustment disorder of 11% (independent of other diagnoses).

The second part of this project aims to investigate the effectiveness, cost-utility and budget impact of the tailored psychological intervention. It is expected that this psychological intervention will be especially effective, since the intervention is tailored to the individual needs regarding intensity of the intervention (i.e. number of modules provided) and wishes regarding the type of intervention. Besides being effective, it is hypothesized that offering this intervention will be cost-effective and potentially even cost-saving.

The cost saving potential is related to the design of the study in which patients are first provided with the short psychological treatment (module 1). The following modules (i.e. module 2 and 3) will only be offered to the patient if the previous psychological treatment module is insufficiently effective. This principle is comparable to a previously investigated stepped care intervention targeting head and neck cancer and lung cancer patients with psychological distress which was shown to be more effective and less costly compared to usual care [17, 27].

### Strengths and limitations

A strength of this study is that its design is in line with clinical practice, as we will investigate a tailored psychological intervention, which may consist of different types of interventions, and not only one specific intervention. Second, this study investigates not only the effectiveness and cost-utility of tailored psychological treatment, but also the prevalence of adjustment disorder and reach of the tailored care. Insight in the prevalence and reach will enable accurate budget impact analysis of providing such psychological treatment to cancer patients with an adjustment disorder. Third, the proposed study will assess the cost-utility and budget impact of the intervention from a healthcare, societal and insurer perspective. So far, the majority of the performed economic evaluations have used the healthcare perspective. However, several guidelines recommend using the societal perspective which includes also for examples productivity losses and informal care costs [61, 62]. This is of importance since these costs have shown to be a great contributor to costs of cancer [63]. Finally, detailed analysis will be conducted on determinants of the prevalence of an adjustment disorder and the reach of psychological care, which will help to identify possible risk groups in the future.

This study, however, also has some limitations. First, this study includes a short follow-up of 6 months in total, which hampers the possibility to investigate the effectiveness and cost-utility of the intervention on the long term. However, a longer waiting list period was considered as not ethical for the patients. Second, the provided intervention consists of a great diversity of evidence-based interventions. Although this approach follows current routine psychological care, it makes it harder to draw conclusions on the effectiveness of any one specific intervention (e.g. self-management or group therapy). Third, this study targets a heterogeneous study population of mixed cancer patients treated with either curative or palliative treatment, which may limit the ability to draw firm conclusions on the effectiveness of psychological treatment in specific study populations. Fourth, as mentioned above a different method will be used to diagnose adjustment disorder compared to methods used in the previous studies, which makes comparison with previous studies harder. However, to investigate the quality of the diagnostic interviews, these interviews will be checked on robustness by audio recording it.

### Implementation and clinical practice

In the Netherlands, psychological treatment for cancer patients undergoing medical treatment is reimbursed as part of the reimbursement of cancer treatment. Since 2012, however, psychological cancer treatment during follow-up (after active cancer treatment) is no longer reimbursed for cancer patients with an adjustment disorder To make an evidence-based decision on future reimbursement of psychological treatment targeting cancer patients with an adjustment disorder, the Dutch minister of Health, Welfare and Sport requested this study on the prevalence of adjustment disorders, actual reach, effectiveness, cost-utility and budget impact of psychological treatment for this patients group. If this study will demonstrate that the psychological intervention is effective and cost-effective, further steps need to be taken to support reimbursement and implementation of this program in clinical practice. In addition, guideline committees will be informed and recommended to adapt the guidelines of tailored psychological care for cancer patients with an adjustment disorder.

## Conclusion

In conclusion, if the psychological intervention is effective and cost-effective, this study will provide support for the reimbursement of psychological interventions for cancer patients with an adjustment disorder. Consequently, this study may contribute to the implementation and optimization of accessibility of psychological treatment for cancer patients with an adjustment disorder. However, considering a broader perspective, this study may also add important knowledge to the literature of economic evaluations of psychological interventions for cancer patients in general.

## Trial status

This study is ongoing. No publications containing the results of this study have been published or submitted to any other journal.

## Data Availability

Full dataset and statistical code will be made available in a repository 3 months after publication of all study outcomes in peer-reviewed journal.

## Abbreviations

CIS: Checklist Individual Strength
DT: distress thermometer
EORTC QLQ-C30: European Organization for Research and Treatment of Cancer Quality of Life Questionnaire
HADS: Hospital Anxiety and Depression Scale
HCP: healthcare professional
iMCQ: Medical consumption questionnaire
iPCQ: Productivity cost questionnaire
MAC: Mental Adjustment to Cancer
MFI: multidimensional fatigue inventory
